# The Silver Lining of Posttraumatic Growth around the Dark Side of the COVID-19 Pandemic: A School-Based Intervention with Mindfulness and Character Strengths Practices among Children

**DOI:** 10.3390/healthcare12020283

**Published:** 2024-01-22

**Authors:** Alexandra Tamiolaki, Argyroula Kalaitzaki, Maria Papadakaki, Elias Kourkoutas

**Affiliations:** 1 Department of Social Work, School of Health Sciences, 71410 Heraklion, Greece; akalaitzaki@hmu.gr (A.K.); mpapadakaki@hmu.gr (M.P.); 2 Laboratory of Interdisciplinary Approaches to the Enhancement of Quality of Life, Hellenic Mediterranean University, 71410 Heraklion, Greece; 3 Laboratory of Health and Road Safety, Hellenic Mediterranean University, 71410 Heraklion, Greece; 4 Department of Primary Education, Faculty of Educational Sciences, University of Crete, 74100 Rethymno, Greece; eliaskourk@uoc.gr

**Keywords:** children, pandemic, mental health, posttraumatic growth, wellbeing, mindfulness, character strengths

## Abstract

The theory of posttraumatic growth (PTG) proposes that from life difficulties positive changes can happen, such as deepened personal relationships and an awareness of new possibilities in life. PTG can occur naturally or can be facilitated. This study aimed to promote PTG through a school-based intervention of eight sessions of 45 min each with mindfulness and character strengths practices (the so-called “The exploration of happiness during the COVID-19 pandemic”). The study conducted assessments at baseline, post-intervention, and follow-up (i.e., one month after the intervention). The post-intervention results showed that the participants in the intervention group experienced an improvement in PTG, well-being, mindfulness, strengths use, and PTS symptoms compared to the children in the control group. Furthermore, these positive changes were sustained at follow-up. The findings of this study highlight that mindfulness-based strengths practices can increase positive outcomes (i.e., well-being, posttraumatic growth) and reduce negative psychological symptoms (PTS) among children. The implications for theory and practice are discussed, and detailed appendices for practitioners are provided.

## 1. Introduction

During the last three years, individuals and communities around the world have faced a challenging period due to the global spread of the coronavirus, known as COVID-19. The restrictive measures to prevent the spread of the virus were the only solution humanity had, but it was also the reason that the world came to know the dark side of being constrained in closed spaces and being devoid of human touch [1]. 

Childhood is a phase in one’s life where social behaviours develop and societal bonding comes into play [1]. Due to the pandemic, children were devoid of it. Entire schooling systems closed, and for a long time, parents kept their children home. While many education systems were able to support learning from home during lockdown periods, students were nonetheless physically isolated [2]. According to Kohlboeck et al. [3], children were particularly vulnerable during this global crisis. The closure of schools, the loss of in-person learning, the separation from friends, peers, teachers, and favourite activities, the excessive information about COVID-19, the fear of contracting the virus, and the substantial changes to their routines may have been interpreted as threatening experiences for many of them [4]. The COVID-19-related measures had a profound effect on their mental health and well-being, and for some, the impact will be lifelong [3,4]. As child mental health is one of the most important issues in the Sustainable Development Goals (SDGs; United Nations [5]), the protection and maintenance of children’s well-being requires special attention. 

Several reviews have examined the negative psychological impact of the COVID-19 pandemic on children’s mental health [6,7]. Among the most severe mental health problems are posttraumatic stress (PTS), such as intrusive thoughts, strong negative feelings (e.g., fear, horror, anger), and nightmares [8]. According to the Diagnostic and Statistical Manual of Mental Disorders, fifth edition (DSM-5), the diagnosis of PTSD requires, in addition to these symptoms, exposure to a traumatic event, defined in criterion A as direct or indirect exposure to death, serious injury, or sexual violence. However, some researchers [9] have argued that criterion A should be expanded to include the COVID-19 pandemic as a traumatic event based on the high rates of PTS, along with the strict quarantines and restrictive measures. As the coronavirus disease continues to circulate, the strict application of DSM-5 criterion A carries the risk of leaving a large number of patients without the appropriate care. Research also suggests that there is a silver lining to many threatening experiences and that negative occurrences such as COVID-19 can be an opportunity for positive psychological changes [10]. These positive changes have been conceptualised under the term posttraumatic growth (PTG), which is defined as a personal transformation resulting from coping with very difficult life crises [11]. PTG occurs after individuals reframe their experiences, change the way they see the world, and perceive potential benefits from life challenges [12]. PTG is manifested in a variety of ways, including an increased sense of personal strength, an appreciation for life, more meaningful interpersonal relationships, the perception of new possibilities and priorities, and a richer existential and spiritual life [11]. During the COVID-19 pandemic, few studies examined PTG among children and adolescents [13,14]. According to researchers, children and adolescents experienced moderate to high levels of PTG [14,15]. Studies have demonstrated that the rates of PTG varied from 9.6% to 45.6% [13,14,15]. These findings indicate that despite the negative impact of COVID-19, positive results are also possible. However, a question arises: under what conditions might children report PTG during the pandemic? 

Researchers [12] have indicated that PTG can occur naturally for some people, and there have been some investigations [16] into individual and environmental factors supporting this transformation. Of great interest is whether PTG can be facilitated. According to Shiyko et al. [17], mindfulness-based interventions (MBIs) can play an important role in the development of PTG and alleviating psychological symptoms (PTSs). These interventions are based on the principle of mindfulness, defined as moment-to-moment, present-cantered, purposeful nonjudgmental awareness [18]. Previous research has found a relationship between mindfulness practice and the majority of PTG aspects. Mindfulness has been linked with spirituality [19], personal strength [20], and improved relationships [21], as well as a heightened appreciation of life [22]. These findings suggest that higher levels of mindfulness predict greater levels of PTG. However, to the best of our knowledge, the limited existing data on MBIs have shown potential benefits mostly in adult cancer patients [23,24]. There is a need to investigate the efficacy of MBIs in the development of PTG in non-clinical populations, such as children, particularly during the COVID-19 crisis.

Character strengths interventions (CSIs) can also play an important role in the development of PTG [25]. These interventions are based on character strengths, defined as 24 positive trait-like capacities for thinking, feeling, and behaving in ways that benefit oneself and others [26]. The 24 character strengths (such as zest, hope, love, and kindness) can be classified into six broad virtue categories (wisdom and knowledge, humanity, courage, transcendence, temperance, and justice) [26]. Previous evidence [27,28] has demonstrated that the virtues of wisdom and knowledge, courage, humanity, and transcendence were most strongly associated with PTG before COVID-19. During the current pandemic, Yu et al. [25] revealed that a CSI caused significant improvements in PTG and well-being. To date, however, the majority of studies have examined the efficacy of CSI in the development of PTG among adults. To our knowledge, no studies have examined the effectiveness of CSIs on PTG among children. Moreover, as individuals can obtain benefits from their character strengths when they make use of them [29], research should emphasise children’s use of their strengths through CSIs. 

A few studies have shown that mindfulness practice and character strengths can be successfully integrated into one intervention (mindfulness-based strength practice, MBSP) [30,31,32]. It is believed that integrating these two practices into one intervention is likely to amplify the positive effects of each one [30]. Mindfulness can cultivate certain character strengths and improve their balanced use [20,32], whereas the use of certain character strengths can improve mindfulness [30,33]. Studies in which these two practices are combined have revealed significantly increased well-being among adolescents [34] and adults [30] and less stress [35]. 

At the time of writing this paper, no study has examined the effectiveness of MBSP on PTG in Greece. The majority of the studies in Greece have focused more on character strengths practices and less on mindfulness [36,37] (although both have positive effects on children’s mental health (such as wellbeing and positive emotions). Therefore, this study aimed to investigate whether a school MBSP intervention called “The exploration of happiness during the COVID-19 pandemic” can facilitate PTG and wellbeing, decrease PTS symptoms, and promote mindfulness and strengths use. The findings will inform social workers and psychologists about the benefits of MBSP as a tool that can be taught and used for promoting and enhancing children’s mental health.

## 2. Materials and Methods

### 2.1. Design 

To analyse the effectiveness of the school programme called “The exploration of happiness during the COVID-19 pandemic” in Greek children, a three-phase structure was used: pre-intervention (baseline), post-intervention, and follow-up research. Twenty Greek schools located in the town of Heraklion in Crete participated in the study. Schools were selected to have similar social backgrounds and were randomly assigned to the intervention (ten schools) or control group (ten schools). The participants in the intervention group received the school programme of interest, whereas the participants in the control group received no intervention. Ethics approval was obtained from the Research Ethics Committee of the Hellenic Mediterranean University. Approval was also granted by the Greek Ministry of Education.

### 2.2. Study Participants

**Figure 1:**
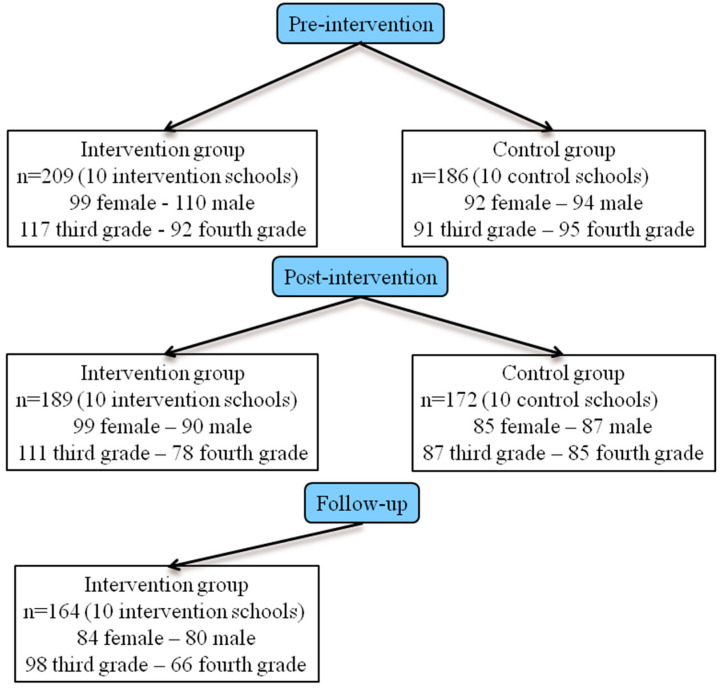
Participants at the pre-intervention, post-intervention, and follow-up stages.

A total of 395 Greek participants of both genders (193 males and 202 females) aged between 8 and 10 years (3rd- and 4th-grade students in primary school) living in Heraklion, Crete, took part in this study. There were 209 participants in the intervention group and 186 in the control group. As for the demographic characteristics of the participants, there were no statistically significant differences in terms of gender (χ^2^ (1, n = 395) = 0.40, *p* = 0.53) or school grade (χ^2^ (1, n = 395) = 1.97, *p* = 0.16) between the two groups. During the study, some participants withdrew for personal reasons or failed to return the self-rated questionnaires. Figure 1 provides an overview of the demographic characteristics of the participants in both the intervention and control groups and the number of participants at the pre-intervention/baseline, post-intervention, and follow-up stages. 

### 2.3. Procedure

The present study was conducted during the school year 2022–2023 in Greek primary schools in Heraklion, Crete. During phase 1, 23 Greek primary schools from 51 were recruited for the study via email and by contacting the board of directors at individual schools, and 20 of them accepted the invitation. Informed consent forms were given to the third- and fourth-grade pupils and their parents to inform them about the study and their rights as participants (e.g., protection of voluntary participation, anonymity, etc.). The parents who consented for their children to participate in the study signed and returned the form to their child’s school. Only Greek children who spoke and understood the Greek language and whose parents provided explicit consent were included in the study. A cross-sectional survey using paper-and-pencil self-rated questionnaires followed to assess PTS symptomatology, wellbeing, PTG, strengths use, and mindfulness among the children. The participants completed the surveys in their classrooms after school hours. No financial incentive was provided. Soon after the intervention and one month later, participants in both groups (intervention and control) completed the same questionnaires again to examine the efficacy of the intervention and if any effects were maintained.

### 2.4. “The Exploration of Happiness during the COVID-19 Pandemic” Intervention

The school-based intervention “The exploration of happiness during the COVID-19 pandemic” was inspired by the MBSP programme developed by Niemiec [31]. It was, however, modified to account for the children’s needs and school timetables. It incorporates developmentally appropriate activities for children. For example, meditations are shorter, and children learn by being actively engaged in lessons rather than listening to an instructor explain the outcomes of mindfulness practice. The eight consecutive sessions per week of 45 min each were embedded in the school curriculum during normal school hours and were held in classrooms. Each group consisted of approximately 15 children. The practitioner of the intervention was an experienced individual with mindfulness training certification from ISON Psychometrica (https://ison.gr/en/ accessed on 20 August 2022). During the first session, the participants learned about mindfulness and a mindfulness pause exercise. Sessions two to five emphasised specific virtues; participants learned the meaning of each virtue and its character strengths, explored through stories how they or others use these virtues and strengths in everyday life (strengths in action story, strengths spotting) [31], and used these virtues and character strengths in activities of mindfulness (acting from strength and mindfulness, strong mindfulness) [31]. During the sixth session, the participants learned that character strengths and virtues can be either overused or underused, and, for that, they used mindfulness to enable them to use character strengths effectively (character strengths use) [31]. During the last two sessions, the participants were guided to make plans for the future in order to live with increased mindfulness and strengths use and to search for growth opportunities through life adversities. An outline of the intervention, examples of the intervention-specific activities, and the internal session structure are presented in Table 1.

### 2.5. Instruments

The demographic characteristics of the students were collected (i.e., gender, grade level), and the following self-report instruments were completed in the pre-test, post-test, and follow-up assessments. 

The Revised Post-Traumatic Growth Inventory for Children (PTGI-C-R) [38] was administered to measure the children’s PTG. Adapted from the PTGI, 10 out of the 21 original items allocated in five subscales (Relating to Others, New Possibilities, Personal Strength, Spiritual Enhancement, and Appreciation of Life) were selected for the PTGI-C-R considering their suitability for children. The participants were instructed to respond in terms of the change that occurred following the COVID-19 pandemic using a 4-point rating ranging from 0 (not at all) to 3 (very much). A higher score indicated higher PTG and greater positive changes. Example items are “I learned how helpful people can be” and “I can handle big problems better”. Cronbach’s alpha for the total scale in this study was 0.91.

The Child PTSD Symptom Scale Self-Report Version for the DSM-5 (CPSS-SR-5) was administered to assess the severity of the children’s PTSD symptoms presented in the past month [39]. The inventory comprises 20 items corresponding to PTSD symptoms according to the four criteria of the DSM-5, namely intrusion, avoidance, negative alterations in cognition and mood, and alterations in arousal and reactivity. The participants rated the frequency with which they experienced each symptom using a 5-point Likert scale, ranging from 0 (never) to 4 (6 or more times per week/almost always). The total severity score ranges from 0 to 80 and is calculated by summing the ratings of the 20 items. Example items are “Having feelings in your body when you remember what happened (for example, sweating, heart beating fast, stomach or head hurting)” and “Having bad dreams or nightmares”. Cronbach’s alpha for the total scale in this study was 0.87.

The Child and Adolescent Mindfulness Measure (CAMM) [40] was used to assess the children’s level of mindfulness. This instrument consists of 10 items assessed on a 5-point Likert scale ranging from 0 (never) to 4 (always). All items are scored in reverse, with higher total scores indicating higher levels of mindfulness. Example items are “At school, I walk from class to class without noticing what I’m doing” and “I think about things that have happened in the past instead of thinking about things that are happening right now”. Cronbach’s alpha for the total scale in this study was 0.92.

The World Health Organization Five Well-being Index (WHO-5) [41] was used to assess the children’s mental well-being over the last two weeks. This instrument consists of 10 items assessed on a 6-point Likert scale ranging from 0 (none) to 5 (always), with higher total scores indicating greater levels of mental well-being. Example items are “I have felt calm and relaxed” and “I have felt active and rigorous”. Cronbach’s alpha for the total scale in this study was 0.90.

The Strengths Use Scale (SUS) developed by Govindji and Linley [42] was used to assess the children’s active use of their strengths. The 14-item questionnaire measured strengths use on a 7-point Likert scale ranging from 1 (strongly disagree) to 7 (strongly agree). Example items are “I achieve what I want by using my strengths” and “Most of my time is spent doing things that I am good at doing.” Cronbach’s alpha for the total scale in this study was 0.98.

### 2.6. Statistical Analyses

Cronbach’s alpha coefficient assessed the internal consistency of the scales. The results of quantitative variables were reported as means and standard deviations (SD). First, a mixed-design MANOVA was used to test the overall differences between the two groups (i.e., the intervention and control groups) at baseline (T1) and after the intervention (T2). Then, two repeated-measures MANOVAs were conducted to compare the effect of the intervention on PTG, wellbeing, PTS, mindfulness, and strengths use at baseline (T1), after the intervention (T2), and at the one-month follow-up (T3) separately in the intervention group and the control group. If an interaction effect between time and group was confirmed, the simple effect was tested to further investigate the results. Following this, post hoc tests were conducted to estimate the specific between-group differences and within-group differences at each time point. A mixed-design MANOVA was also used to test the differences between gender and grade on PTG, wellbeing, PTS, mindfulness, and strengths use at baseline (T1), after the intervention (T2), and at the one-month follow-up (T3) in the intervention group. Furthermore, three multiple linear regression analyses (using the enter method) were conducted to investigate the potential effect of mindfulness and strengths use on PTG, wellbeing, and PTS after the intervention (T2) and at the one-month follow-up (T3) separately. The significance level was set at *p* < 0.05. The measure of the effect size was the partial eta-squared value (η^2^). All data analyses were performed using SPSS version 20.0 (IBM Co., Ltd., Chicago, IL, USA).

## 3. Results

The results of the mixed-design MANOVA showed that there were statistically significant overall differences between the two groups (i.e., the intervention and control groups) (Wilk’s Λ = 0.56; F (5; 355) = 56.56; *p* < 0.001; η^2^ = 0.44), within timepoints T1 and T2 (Wilk’s Λ = 0.31; F (5; 355) = 155.80; *p* < 0.001; η^2^ = 0.69), and within the time × group interaction (Wilk’s Λ = 0.30; F (5; 355) = 164.63; *p* < 0.001; η2 = 0.70). Specifically, the participants reported statistically significant differences in the time x group interaction in terms of PTG (F (1, 359) = 627.54; *p* < 0.001; η^2^ = 0.64), wellbeing (F (1, 359) = 452.24; *p* < 0.001; η^2^ = 0.56), PTS (F (1, 359) = 197.88; *p* < 0.001; η^2^ = 0.36), mindfulness (F (1, 359) = 475.06; *p* < 0.001; η^2^ = 0.57), and strengths use (F (1, 359) = 618.85; *p* < 0.001; η^2^ = 0.63). The between-group post hoc tests (ANOVA) showed that there was no significant difference in the study variables between the two groups (intervention and control) at T1. Significant differences were found between the groups at T2. The participants in the intervention group scored significantly higher than the participants in the control group in all variables (i.e., PTG, wellbeing, mindfulness, strengths use, and PTS) at T2 (see Table 2). These results underline the effectiveness of the MBSP intervention in improving participants’ PTG (both total and subscales’), well-being, mindfulness, strengths use, and PTS. 

The repeated-measures MANOVA yielded a significant main effect of time in the intervention group (Wilk’s Λ = 0.15; F (10, 644) = 99.35; *p* < 0.001; η^2^ = 0.61) but not in the control group (Wilk’s Λ = 0.98; F (5, 167) = 0.61; *p* = 0.69; η^2^ = 0.02) on the development of the variables within a group (PTG, wellbeing, mindfulness, strengths use and PTS). Specifically, for the intervention participants, a significant main effect of time was reported on PTG (F (2, 326) = 659.26, *p* < 0.001, η^2^ = 0.80), wellbeing (F (2, 326) = 527.09; *p* < 0.001; η^2^ = 0.76), PTS (F (2, 326) = 227.39; *p* < 0.001; η^2^ = 0.58), mindfulness (F (2, 326) = 514.70; *p* < 0.001; η^2^ = 0.76), and strengths use (F (2, 326) = 635.18; *p* < 0.001; η^2^ = 0.80). The within-group post hoc results showed that the intervention participants scored significantly higher in all variables (i.e., PTG, wellbeing, mindfulness, strengths use, and PTS) at T2 compared to T1. No significant differences were reported between T2 and T3 (one month after the intervention). These findings indicated that the MBSP intervention could enhance the overall PTG and PTG subscales’ scores, wellbeing, mindfulness, strengths use, and PTS, and that the positive results remained at T3 (see Table 3). In the intervention group, the regression analyses showed that mindfulness and strengths use were predictors of PTG, wellbeing, and PTS (see Table 4). 

The results of the mixed-design MANOVA also showed that there were statistically significant differences in the intervention group between males and females (Wilk’s Λ = 0.91; F (5, 158) = 3.06; *p* < 0.05; η^2^ = 0.09) for all the study variables (PTG, wellbeing, mindfulness, strengths use, and PTS). However, there were no statistically significant differences within the time × gender interaction (Wilk’s Λ = 0.96; F (5, 153) = 0.62; *p* = 0.79; η2 = 0.04) at T1, T2, and T3. The results for each variable concerning the gender of the students are depicted in Table 5. These findings underline the effectiveness of the MBSP intervention in improving participants’ PTG, well-being, mindfulness, strengths use, and PTS for both genders.

The results of the mixed-design MANOVA in the intervention group also showed that there were statistically significant differences between the third- and fourth-grade students (Wilk’s Λ = 0.83; F (5, 158) = 6.62; *p* < 0.001; η^2^ = 0.17) for all the study variables (PTG, wellbeing, mindfulness, strengths use, and PTS). The results for each variable concerning the third- and fourth-grade students are depicted in Table 6. Furthermore, there were statistically significant differences within the time × grade interaction (Wilk’s Λ = 0.81; F (5, 153) = 3.44; *p* < 0.001; η^2^ = 0.18). The univariate tests of the mixed-design MANOVA showed a significant difference within the time × grade interaction only for mindfulness. These findings suggest that the MBSP intervention was more effective in increasing mindfulness for the participants in the third grade than those in the fourth grade (see Table 5).

## 4. Discussion

According to researchers, there is a silver lining to the COVID-19 experience [10]. Increasing evidence among children suggests that the COVID-19 pandemic could be an opportunity for positive life changes in PTG [13,15]. However, to the best of our knowledge, no intervention has been specifically designed to facilitate the development of PTG among children during this public health emergency. Moreover, less is known about the practices that promote PTG among children. In order to fill this gap, we examined the efficacy of a positive psychology school intervention based on character strengths and mindfulness practices targeting PTG development, wellbeing improvement, and PTS decrease. 

The findings of the study provide evidence that an 8-week school intervention programme with mindfulness-based strengths practice (MBSP) significantly improved PTG for the intervention participants compared with a control group with no intervention. Although at the pre-intervention assessment, both the participants of the intervention and the control group experienced low or no PTG, at the post-intervention assessment, a significant increase in PTG scores and a stabilisation of the scores at the follow-up (one month after the intervention) occurred. More specifically, after the MBSP intervention, the participants experienced increased PTG in all five dimensions, thus having closer relationships with others, a greater sense of personal strength, increased appreciation for their lives, increased spiritual development, and a recognition of new possibilities or paths for their lives. Thus, it can be assumed that through MBSP, healthcare workers and potentially teachers could help children promote PTG and perceive the difficult moments in their lives as learning experiences and growth opportunities.

In relation to the five PTG subscale dimensions, the intervention participants experienced greater improvements in “Relating to others” and “New possibilities”. The improvement in relationships is in line with the existing literature among adults that underlines the effectiveness of the combination of mindfulness and character strengths practices in valuing relationships [35]. According to this study [35], individuals who participated in a MBSP were more patient in their interactions with others or able to better understand others. The high scores for the dimension of “New possibilities” are a substantial finding, indicating that the emphasis through MBSP activities on future goals and plans enabled the participants to develop new interests or a new life path and a willingness to change things that need change and helped them recognise new opportunities through the COVID-19 adversity. 

In line with our expectations, it was found that MBSP significantly increased well-being and decreased symptoms of PTS. At the pre-intervention assessment, both the participants in the intervention and the control group experienced low levels of well-being and moderate levels of PTS. At the end of the programme, the intervention participants experienced a significant improvement in well-being and PTS compared to the control group, which remained at the one-month follow-up. Previous research among adults suggested that MBSP has a positive impact on adults’ well-being [30,34,43] and stress reduction [35]. The present study expands the current knowledge underlining the beneficial effects of MBSP on children’s mental health. 

It was also found that MBSP had a significant effect on the intervention participants from pre-intervention to post-intervention on promoting mindfulness and strengths use and a stabilisation effect at the follow-up. These positive improvements were not observed in the control group. An increase in mindfulness and character strengths through MBSP has been previously shown among adults [30,31,32]. The results replicate and extend the existing literature, indicating that MBSP is successful in teaching and developing both mindfulness and strengths use among children. Further to this, the present study demonstrates the predictive role of mindfulness and strengths use on PTG, well-being, and PTS. The findings revealed that mindfulness and strengths use have a buffering effect on PTS (prevent negative psychological symptoms), a bolstering effect on well-being (promote mental health), and a building effect on PTG (create growth opportunities). In support of this notion, mindfulness and strengths use could be considered two different but connected pathways that serve as a buffer or source of protection for mental health. It seems that they create a positive synergy of mutual benefit and help children to heal, overcome, and bounce forward from adversity. This synergy might actually be what underlies successful school-based positive psychology interventions. Beyond involving social workers and school psychologists in school-based MBSP interventions, we further encourage studies that examine mindfulness and strengths use in the field of school psychology. This is essential, particularly given the vulnerable nature of the child population during humanitarian crises such as pandemics [44] and the potentially lasting impact of school-based interventions.

Another finding worth noting is that although females experienced higher levels of PTG, wellbeing, mindfulness, and strengths use and lower levels of PTS than males, the MBSP intervention increased PTG, wellbeing, mindfulness, and strengths use and decreased PTS in the participants of both genders. Previous school interventions based on mindfulness [45] or character strengths [46] have shown that these programmes were more effective for females than males, and researchers [45,47] recommended future interventions to be gender-specific. The findings of this study suggest that the present MBSP intervention could be equally effective on both genders for promoting children’s mental health. However, it was found that the MBSP intervention differently affected mindfulness for the fourth-grade and third-grade students. Although the fourth-grade students experienced higher levels of PTG, wellbeing, mindfulness, and strengths use and lower levels of PTS than the third-grade students, in line with previous studies [48,49,50], through the MBSP intervention, the third-grade children experienced greater improvements in mindfulness than the fourth-grade children. In agreement with Sheinman et al.’s [51] mindfulness-based school intervention, the younger children experienced a significantly greater advancement in mindfulness than the older children. It could be that the mindfulness techniques of the present MBSP intervention were more appealing for the third-grade children than those in the fourth grade. Cairncross and Miller [52] suggested that mindfulness techniques that use active movement or visualisation may be more favourable for younger children than older ones. Future research may need to explore the effects of different mindfulness techniques for different grade levels or age groups in order to develop a greater understanding of how mindfulness training works to promote the observed benefits and inform the design of future MBSP programmes in the school context.

Several limitations should be considered. First, the use of self-report measures may have led to response bias. Response bias can be minimised by using a combination of self-report measures with observations, interviews, etc. [53]. The intervention and control schools of the present study were located in the same area, and thus the generalisation of the findings to all Greek children is not possible. Although the sample size in this study was relatively satisfactory, a larger sample of people of different ages, cultural or social backgrounds, and educational levels would be necessary to generalize the results. The participants in the intervention group consisted mainly of children with moderate levels of PTS, and there is no certainty that the intervention exercises are suitable for children with more difficulties. The control group did not participate in the follow-up assessment. Finally, future studies with longer follow-ups should be conducted to better understand the long-term effects of the intervention. 

Despite its limitations, this study has several strengths. The programme “The exploration of happiness during the COVID-19 pandemic” is the first longitudinal study to assess the effectiveness of MBSP in children. It enriches the existing literature on the combination of mindfulness and character strengths activities (i.e., the integrated MBSP) and provides theoretical support for this integrated MBSP among children. Furthermore, it is the first intervention that investigates the association between MBSP and PTG and MBSP’s contribution to promoting PTG. Moreover, it sheds light on the role of MBSP in enhancing well-being, mindfulness, and strengths knowledge and use and reducing PTS.

This study also has important practical implications. As the present MBSP activities were beneficial for the children, educational and mental health institutions should organise MBSP training programmes to better support children’s mental health, particularly during a pandemic. Healthcare workers (HCWs) could implement mindfulness and character strengths programmes not only within school settings but also online through e-learning courses and websites. HCWs could organise those MBSP programmes not only for children who are experiencing the COVID-19 crisis but also for those who experience other life adversities such as natural disasters (earthquake, hurricane), community or school violence, and parental divorce. Also, it would be helpful if HCWs could make all of the MBSP activities deaf- or blind-friendly with simple and small adaptations. Furthermore, MBSP training programmes, seminars, and workshops for HCWs, parents, and teachers could potentially have a synergistic effect on children’s mental health. Finally, this study provided cost-effective intervention materials that can be easily implemented. 

## 5. Conclusions

The present school-based intervention provides clear evidence that MBSP is a tool that can be taught (to increase mindfulness and strengths use) and used to promote PTG and overall mental health (increase wellbeing and decrease PTS) among children. The take-home message of the programme “The exploration of happiness during the COVID-19 pandemic” could be that mindfulness and character strengths can help participants to grow through life and not just go through life. Mindfulness and character strengths were shown to be important pathways for helping children see life difficulties as opportunities to become stronger or as stepping stones towards a more empowered self. Through the use of mindfulness and character strengths, children could realise that even the most challenging experiences can serve as catalysts for self-improvement and personal development. Even though there are very few studies to date to support the effectiveness of MBSP, our research findings are promising and offer unique perspectives that warrant future investigation.

## Figures and Tables

**Table 1:** Outline of the intervention, indicative session activities, and internal session structure.

Session Topic	Main Idea	Exercises	Description
1. Mindfulness	Mindful or Mindfull?	Mindfulness pause through mindfulness jar	The autopilot mind is pervasive; change opportunities start with attention in the present moment.
2. Virtues and character strengths	The Virtue of Wisdom	You at your best	Remember a day that you used the virtue of wisdom and succeeded.
		Character strengths breathing space	Use the virtue of wisdom and learn how to relax through a mindfulness breathing exercise.
3. Difficulties are opportunities	The Virtue of Courage	Inspiring stories of courage	Describe how the hero in the story expresses his courage.
		Courage film (mindful watching and character strengths)	Use the virtue of courage and carefully watch wild animals. Leave behind your thoughts as you dive deeply into looking and seeing.
4. Embracing the beauty of every moment	The Virtue of Transcendence	A moment of appreciation	Describe something you felt was beautiful that is from nature.
		Sounds collectors (mindful hearing)	Use today’s virtue and appreciate the unique sounds of nature.
5. Opportunities for personal and relationship growth	The Virtue of Humanity and Justice	Inspiring stories of humanity and justice	Describe how the hero in the film expresses the virtue of humanity and justice.
		Actions of love (mindful living)	Use today’s virtues and share a meal with others.
6. Mindfulness of the Golden Mean	The Virtue of Temperance	Reframing	Mindfulness helps to use character strengths more properly.
7. Future dreams and goals	Being the best you and the best person you can be	Best possible self	Focus on a future goal and think which character strengths you should use to achieve it.
8. A rainbow of growth opportunities awaits	Taking stock of what has been learned and how to be proactive in keeping up the practice	Sacred object meditation: after the storm comes the rainbow	Make a rainbow paint to remember that there is always a rainbow after every storm, and something beautiful happens after difficult times.
**MBSP General Internal Session Structure**			
1. Opening meditation with a mindfulness pause and the use of the mindfulness glitter jar.			
2. Discussion about the out-of-previous-session exercises.			
3. Participants focused on a virtue and its character strengths and used them through a mindfulness exercise (strong mindfulness), or, in turn, participants used mindfulness as a lens for deepening awareness and use of character strengths (character strengths use).			
4. Suggested homework exercises for the next session.			
5. Closing meditation with a mindfulness pause and the use of the mindfulness glitter jar.			

**Table 2:** Results of between-group post hoc tests and ANOVAs showing means, F, and effect sizes (η^2^) at baseline and after the intervention.

	Baseline (T1)					After the Intervention (T2)				
	Intervention Group	Control Group	F (1, 394)	*p*	η^2^	Intervention Group	Control Group	F (1, 360)	*p*	η^2^
	M (SD)	M (SD)				M (SD)	M (SD)			
PTS ^a^	25.71 (11.53)	24.51 (9.82)	1.23	0.27	0.003	14.65 (8.87)	24.42 (10.95)	87.56	<0.001	0.20
WHO ^b^	11.65 (4.60)	11.38 (4.60)	0.34	0.56	0.001	18.79 (4.86)	11.38 (5.19)	196.41	<0.001	0.35
CAMM ^c^	17.89 (9.58)	16.23 (8.93)	3.13	0.08	0.008	32.84 (6.78)	16.38 (9.40)	368.39	<0.001	0.51
PTGI ^d^	11.18 (5.05)	11.51 (5.13)	0.40	0.53	0.001	22.60 (6.55)	11.51 (5.68)	293.38	<0.001	0.45
Relating to others	3.70 (1.71)	3.85 (1.71)	2.24	0.38	0.002	5.29 (1.08)	3.90 (1.66)	89.96	<0.001	0.20
Personal strength	1.34 (1.33)	1.52 (1.36)	3.08	0.19	0.004	4.32 (1.84)	1.54 (1.31)	269.28	<0.001	0.43
Appreciation of life	1.37 (1.40)	1.39 (1.30)	0.03	0.89	0.000	4.38 (1.66)	1.35 (1.43)	340.81	<0.001	0.49
Spiritual change	2.69 (1.69)	2.72 (1.77)	0.04	0.90	0.000	4.07 (1.71)	2.74 (1.67)	55.85	<0.001	0.14
New possibilities	1.93 (1.33)	2.03 (1.23)	0.49	0.59	0.001	4.55 (1.53)	1.97 (1.31)	292.37	<0.001	0.45
SUS ^e^	53.43 (15.39)	52.68 (13.65)	0.26	0.61	0.001	82.06 (16.06)	52.09 (15.73)	319.91	<0.001	0.47

^a^ posttraumatic stress symptoms; ^b^ wellbeing; ^c^ mindfulness; ^d^ posttraumatic growth; ^e^ strengths use.

**Table 3:** Results of within-group post hoc tests and ANOVAs showing means, F, and effect sizes (η^2^) in the intervention group at baseline (T1), after the intervention (T2), and one month after the intervention (T3).

	T1	T2	F (1, 163)	*p*	η^2^	T3	F (1, 163)	*p*	η^2^
	M (SD)	M (SD)				M (SD)			
PTS ^a^	25.36 (11.56)	14.74 (8.43)	227.39	<0.001	0.58	14.55 (8.39)	1.58	0.21	0.010
WHO ^b^	11.87 (4.67)	18.76 (4.68)	559.57	<0.001	0.77	18.71 (4.80)	0.32	0.57	0.002
CAMM ^c^	18.46 (9.61)	32.93 (6.35)	527.86	<0.001	0.76	32.98 (6.46)	0.30	0.59	0.002
PTGI ^d^	11.33 (4.93)	22.70 (6.33)	687.08	<0.001	0.81	22.47 (6.24)	2.80	0.10	0.017
Relating to others	3.74 (1.72)	5.35 (1.02)	148.41	<0.001	0.48	5.43 (0.93)	3.43	0.07	0.021
Personal strength	1.39 (1.36)	4.31 (1.81)	456.11	<0.001	0.74	4.28 (1.67)	0.21	0.65	0.001
Appreciation of life	1.37 (1.43)	4.41 (1.62)	523.55	<0.001	0.76	4.33 (1.55)	1.62	0.21	0.010
Spiritual change	2.80 (1.73)	4.09 (1.70)	71.26	<0.001	0.30	3.99 (1.72)	1.67	0.20	0.010
New possibilities	2.02 (1.29)	4.54 (1.52)	445.02	<0.001	0.73	4.44 (1.54)	1.76	0.19	0.011
SUS ^e^	54.18 (14.92)	82.21 (15.75)	656.08	<0.001	0.80	82.37 (15.98)	0.38	0.54	0.002

^a^ posttraumatic stress symptoms; ^b^ wellbeing; ^c^ mindfulness; ^d^ posttraumatic growth; ^e^ strengths use.

**Table 4:** Regression analyses for predicting PTG, wellbeing, and PTS by mindfulness and strengths use at baseline (T1), after the intervention (T2), and one month after the intervention (T3).

		T1			T2			Τ3		
		B (SE)	Β	T	B (SE)	β	T	B (SE)	β	t
PTGI ^a^	CAMM ^d^	0.37 (0.03)	0.70	10.99 ***	0.37 (0.04)	0.38	9.02 ***	0.38 (0.05)	0.40	7.74 ***
	SUS ^e^	0.06 (0.02)	0.19	2.94 **	0.25 (0.02)	0.60	14.16 ***	0.23 (0.02)	0.58	11.35 ***
WHO ^b^	CAMM ^d^	0.34 (0.02)	0.71	19.28 ***	0.37 (0.03)	0.51	11.92 ***	0.38 (0.04)	0.51	10.08 ***
	SUS ^e^	0.08 (0.01)	0.28	7.60 ***	0.14 (0.01)	0.47	11.02 ***	0.14 (0.02)	0.46	9.06 ***
PTS ^c^	CAMM ^d^	−0.74 (0.08)	−0.61	−9.63 ***	−0.55 (0.06)	−0.42	−8.36 ***	−0.53 (0.07)	−0.40	−7.49 ***
	SUS ^e^	−0.21 (0.05)	−0.28	−4.48 ***	−0.30 (0.03)	−0.55	−10.97 ***	−0.29 (0.03)	−0.60	−10.33 ***

^a^ posttraumatic growth; ^b^ wellbeing; ^c^ posttraumatic stress symptoms; ^d^ mindfulness; ^e^ strengths use. ** *p* < 0.01. *** *p* < 0.001.

**Table 5:** Results of mixed-design MANOVA’s univariate tests for gender differences in the intervention group.

		Τ1	Τ2	Τ3	Time		Gender		Time x Gender	
	Gender	M (SD)	M (SD)	M (SD)	F (2, 324)	η^2^	F (1, 162)	η^2^	F (2, 324)	η^2^
PTS ^a^	Male	27.54 (11.92)	16.73 (8.52)	16.74 (8.88)	226.06 ***	0.58	10.05 **	0.06	3.86	0.000
	Female	23.29 (10.87)	12.86 (7.94)	12.48 (7.36)						
	Total	25.36 (11.56)	14.74 (8.43)	14.55 (8.39)						
WHO ^b^	Male	11.06 (4.03)	17.74 (4.45)	17.64 (4.64)	524.78 ***	0.76	8.01 **	0.05	5.57	0.004
	Female	12.63 (5.12)	19.73 (4.71)	19.74 (4.74)						
	Total	11.87 (4.67)	18.76 (4.68)	18.71 (4.80)						
CAMM ^c^	Male	17.04 (8.49)	31.34 (6.83)	31.29 (7.03)	511.27 ***	0.76	9.38 **	0.06	5.59	0.001
	Female	19.82 (10.44)	34.45 (5.49)	34.60 (5.42)						
	Total	18.46 (9.61)	32.93 (6.35)	32.98 (6.46)						
PTGI ^d^	Male	10.82 (4.28)	21.63 (6.06)	21.23 (5.97)	662.07 ***	0.80	5.15 *	0.03	40.65	0.014
	Female	11.81 (5.47)	23.71 (6.45)	23.65 (6.30)						
	Total	11.33 (4.93)	22.70 (6.33)	22.47 (6.24)						
SUS ^e^	Male	52.01 (14.73)	79.69 (15.82)	79.49 (16.41)	631.48 ***	0.80	5.20 *	0.03	36.60	0.002
	Female	56.25 (14.88)	84.61 (15.39)	85.12 (15.15)						
	Total	54.18 (14.92)	82.21 (15.75)	82.37 (15.98)						

^a^ posttraumatic stress symptoms; ^b^ wellbeing; ^c^ mindfulness; ^d^ posttraumatic growth; ^e^ strengths use. * *p* < 0.05. ** *p* < 0.01. *** *p* < 0.001.

**Table 6:** Results of mixed-design MANOVA’s univariate tests for grade differences in the intervention group.

		Τ1	Τ2	Τ3	Time		Grade		Time × Grade	
	Grade	M (SD)	M (SD)	M (SD)	F (2, 324)	η^2^	F (1, 162)	η^2^	F (2, 324)	η^2^
PTS ^a^	3rd grade	27.44 (12.02)	16.44 (8.20)	16.35 (8.52)	214.58 ***	0.57	12.22 **	0.07	0.35	0.002
	4th grade	22.27 (10.16)	12.23 (8.19)	11.89 (7.50)						
	Total	25.36 (11.56)	14.74 (8.43)	14.55 (8.39)						
WHO ^b^	3rd grade	10.81 (4.26)	17.95 (4.57)	17.80 (4.76)	499.34 ***	0.76	11.78 **	0.07	0.80	0.005
	4th grade	13.44 (4.85)	19.95 (4.62)	20.08 (4.55)						
	Total	11.87 (4.67)	18.76 (4.68)	18.71 (4.80)						
CAMM ^c^	3rd grade	16.15 (8.82)	32.39 (6.12)	32.29 (6.19)	496.48 ***	0.75	8.24 **	0.05	11.10 **	0.064
	4th grade	21.89 (9.78)	33.74 (6.64)	34.02 (6.74)						
	Total	18.46 (9.61)	32.93 (6.35)	32.98 (6.46)						
PTGI ^d^	3rd grade	10.52 (4.67)	21.78 (6.37)	21.54 (6.20)	633.80 ***	0.80	7.22 **	0.04	0.10	0.001
	4th grade	12.53 (5.11)	24.06 (6.06)	23.85 (6.10)						
	Total	11.33 (4.93)	22.70 (6.33)	22.47 (6.24)						
SUS ^e^	3rd grade	49.73 (13.32)	79.07 (15.99)	78.85 (16.52)	601.63 ***	0.79	18.84 ***	0.10	1.63	0.010
	4th grade	60.79 (14.80)	86.86 (14.26)	87.61 (13.66)						
	Total	54.18 (14.92)	82.21 (15.75)	82.37 (15.98)						

^a^ posttraumatic stress symptoms; ^b^ wellbeing; ^c^ mindfulness; ^d^ posttraumatic growth; ^e^ strengths use. ** *p* < 0.01. *** *p* < 0.001.

## Data Availability

The data presented in this study are available on request from the corresponding author.
